# Analyzing the Effects of a G137V Mutation in the *FXN* Gene

**DOI:** 10.3389/fnmol.2015.00066

**Published:** 2015-11-25

**Authors:** Nathalie Faggianelli, Rita Puglisi, Liana Veneziano, Silvia Romano, Marina Frontali, Tommaso Vannocci, Silvia Fortuni, Roberto Testi, Annalisa Pastore

**Affiliations:** ^1^Department of Basic and Clinical Neuroscience, Maurice Wohl Institute, King’s College LondonLondon, UK; ^2^CNR Institute of Translational PharmacologyRome, Italy; ^3^Sant’Andrea Hospital, University of Rome La SapienzaRome, Italy; ^4^Department of Biomedicine and Prevention, University of Rome “Tor Vergata”Rome, Italy

**Keywords:** disease mechanisms, frataxin, friedreich’s ataxia, functional mutants, protein folding

## Abstract

Reduced levels of frataxin, an essential mitochondrial protein involved in the regulation of iron-sulfur cluster biogenesis, are responsible for the recessive neurodegenerative Friedreich Ataxia (FRDA). Expansion of a GAA triplet in the first intron of the FRDA is essential for disease development which causes partial silencing of frataxin. In the vast majority of cases, patients are homozygotes for the expansion, but a small number of FRDA patients are heterozygotes for expansion and point mutations in the frataxin coding frame. In this study, we analyze the effects of a point mutation G137V. The patient P94–2, with a history of alcohol and drug abuse, showed a FRDA onset at the border between the classic and late onset phenotype. We applied a combination of biophysical and biochemical methods to characterize its effects on the structure, folding and activity of frataxin. Our study reveals no impairment of the structure or activity of the protein but a reduced folding stability. We suggest that the mutation causes misfolding of the native chain with consequent reduction of the protein concentration in the patient and discuss the possible mechanism of disease.

## Introduction

Friedreich ataxia (FRDA, OMIM 229300) is the most common form of recessively inherited ataxia, a neurodegenerative disease associated to loss of voluntary movement (Pastore and Puccio, 2013). Although the disease incidence is *ca.* 1 in 50,000 individuals, heterozygotic carriers are as frequent as 1 in 250 individuals. Primarily, FRDA is caused by the presence of a (GAA)n expansion within the first intron of the *FXN* gene, which encodes the frataxin protein. The severity of the disease is correlated with the number of repeats (Pastore and Puccio, 2013), which is lower than 30 in normal alleles and between 66 and 1700 in FRDA patients (Clark et al., 2004). Repeat expansion leads to epigenetic changes which result in partial gene silencing by blocking the transition from initiation to a productive elongation of frataxin transcription (Kim et al., 2011). Typically, FRDA patients have frataxin levels in peripheral tissues that range from 2–30% as compared to control levels (Deutsch et al., 2010; Nachbauer et al., 2011; Saccà et al., 2011, 2013). These levels correlate directly with age of onset and inversely with the number of GAA repeats. Carriers who do not develop symptoms of FRDA have typically frataxin levels from 30–80% of control levels. However, a certain variability is observed which makes it difficult to correlate precisely the frataxin levels with the severity of phenotype.

The vast majority of FRDA patients are homozygous for the expansion. A small fraction of patients (4%), however, are heterozygous for triplets expansion on one allele and a point mutation on the other (Cossée et al., 1999). To date at least 20 point mutations have been reported (Pook et al., 2000). These patients can present either the classical FRDA phenotype or an atypical clinical presentation (Cossée et al., 1999). The genotype-phenotype correlation of such patients is usually less predictable depending on the nature and position of the mutation (Pook et al., 2000). Contrary to GAA expansion that alters protein levels, these mutations often directly affect the function of the expressed protein. The phenotype of these patients is nevertheless most of the time indistinguishable from homozygous individuals, except in the case of a few missense mutations causing atypical or milder clinical presentations (Cossée et al., 1999; Gellera et al., 2007).

A close study of these point mutations, by looking at their effects on the fold, stability and activity of frataxin is interesting to gain new insights into the role of frataxin in the onset of the disease and contribute to a better understanding of the protein function.

Frataxin is ubiquitously expressed at low levels. Higher concentrations are found in the heart, spinal cord and dorsal root ganglia, all tissues that are heavily dependent on oxidative respiration (Campuzano et al., 1996). Frataxin is an acidic protein with iron-binding properties and highly conserved in most organisms from bacteria to mammals (Adinolfi et al., 2002). It is a nuclearly-encoded mitochondrial protein (Campuzano et al., 1997). The mature human protein contains 130 amino acids (Condò et al., 2007) 14kDa distributed in two structurally distinct regions, with a C-terminal globular domain preceded by a flexible and unstructured N-terminus which is part of the mitochondrial import signal (Musco et al., 2000; Prischi et al., 2010). Frataxin is an essential component of the iron-sulfur cluster biogenesis (Mühlenhoff et al., 2003; Adinolfi et al., 2009) in which it acts by interacting with the two central components of this machinery, the desulphurase Nfs1/IscS (human and bacterial forms, respectively) and the scaffold protein Isu/IscU (human and bacterial forms, respectively; Prischi et al., 2010). Independent evidence suggests that frataxin acts as a sensor protein, tuning the quantity of clusters formed in order to match the concentration of the apo-acceptors (Adinolfi et al., 2009).

In this study, we examined a point G137V mutation, found in a FRDA patient. We explored the structure-function relationship and carried out a detailed investigation of the possible consequences of this mutation on the frataxin fold, stability and function using complementary biochemical and biophysical structural techniques *in vitro* which include Circular Dichroism (CD), absorbance and Nuclear Magnetic Resonance (NMR) spectroscopies and enzymatic assays. Structural studies revealed no appreciable consequences on the fold and activity but an appreciable loss of stability. We suggest that reduced stability might affect the efficiency of folding of the native chain resulting in reduced levels of the protein, as also observed for other FRDA clinical mutations.

## Materials and Methods

### Genetic Test

The genetic test was approved by the Ethical Commettee of Azienda Ospedaliera Sant’Andrea of Rome “La Sapienza” and conducted according to the principles expressed in the Declaration of Helsinki. All participants signed written informed consent.

### Mutation Analysis

The test for the specific disease mutation (expansion of GAA repeats in FXN gene LRG_339t1 intron 1) was performed in double by PCR amplification of GAA repeats (Filla et al., 1996). The result was confirmed by TP PCR on capillary electrophoresis (Ciotti et al., 2004). DNA sequencing for mutation screening of FXN gene was performed using primer pairs according to a previously described protocol (Campuzano et al., 1996).

### Expression and Purification of Recombinant Proteins in *E. Coli*

The *QuikChange* method (*Stratagene*) was used to introduce the point mutation into a pET-24 plasmid containing the human frataxin gene (residues δ91–210, corresponding to the mature form of human frataxin hereon called Hfra(91–210)). The mutation site was confirmed by DNA sequencing. All recombinant proteins were expressed in *E. coli* and purified according to previously published protocols (Musco et al., 2000; Prischi et al., 2010). In short, the plasmid containing the variant as a fusion protein with His6 and GST (glutathione S-transferase) tags and a cleavage site for Tobacco Etch Virus (TEC) was transformed into the *E. coli* strain BL21(DE3)pLys (Novagen). The cells were grown in Luria–Bertani (LB) medium with kanamycin. Protein expression was induced with IPTG. The cells were harvested and lysed by sonication. After centrifugation, the supernatant was loaded on Ni-NTA agarose beads (batch) and the protein eluted with 300 mM imidazole. The tag was cleaved with the TEV protease overnight. A second step of Ni-NTA agarose purification allowed collecting the protein eluted separately from the tag. Final purification was performed by gel filtration. The procedure followed for the purification of Nfs1 and Isu was the same except for the further addition of biotine-thiamine (0.001–0.005 mg/mL) and 8.3 mM zinc in the growing medium of Nfs1 and Isu respectively. 0.5 mM TCEP was preferred over DTT in the purification protocol of Isu.

### Circular Dichroism Measurements

Far-UV CD spectra were recorded with a Jasco J-815 spectropolarimeter using a cell holder thermostated by a CDF-426S Peltier unit. All CD measurements were carried out in the buffer used for gel filtration (20 mM Tris-HCl at pH 8, 150 mM NaCl and 2 mM DTT) with 10 μM protein, using fused silica cuvettes with 1 mm pathlength (Hellma Materials GmbH & Co, Jena, Germany). The spectra were typically recorded with 0.1 nm resolution and baseline corrected by subtraction of the appropriate buffer spectrum. Secondary structure content was estimated using methods described by Sreerama and Woody (2000). Thermal unfolding curves were obtained by monitoring the ellipticity at 222 nm using 2 mm path length cuvettes and a heating rate of 1°C/min over the temperature range 0–80°C. The transition mid-point temperature was obtained by fitting to the modified Gibbs-Helmholtz equation using in-house software as described elsewhere (Martin and Schilstra, 2008). Each experiment was repeated atleast three times.

### Nuclear Magnetic Resonance

One-dimensional ^1^H NMR spectra were recorded at 298 K on Varian INOVA spectrometer operating at 600 MHz ^1^H frequency (14.1 Tesla magnetic field). The proteins (100 μM) were dissolved in the same buffer as for CD, with a further addition of 10% deuterated water.

### *In Vitro* Iron-Sulfur Cluster Formation On IscU

Enzyme kinetics experiments were performed in a total volume of 800 μL. The reaction mixtures were prepared under anerobic conditions using a Controlled Atmosphere Chamber (Belle technology). The scaffold protein IscU (50 μM) was pre-incubated with Nfs1 (1 μM), frataxin or its mutant (5 μM), 3 mM DTT and 25 μM iron in buffer (20 mM Tris-HCl at pH 8, 150 mM NaCl) for 30 min. The reaction was initiated by addition of 250 μM L-cysteine. Kinetic curves of the iron-sulfur cluster formation *in vitro* were obtained by following the variation in the CD at 435 nm during the time using a Jasco J-815 CD spectropolarimeter. Each experiment was repeated atleast three times.

### Frataxin Levels

The frataxin levels in the proband, his father and mother were probed by Western blot. A healthy individual was used as a control. The experiment was carried out in triplicates starting from two independent blood samples taken approximately one month apart. Fresh peripheral blood mononuclear cells (PBMC) were isolated from blood by density gradient centrifugation with Lympholyte Cell Separation Media (Cederlane) in Sepmate tubes (StemCell Technologies) following the manufacturer’s protocol. Total cell extracts from PBMC were prepared in ice-cold modified RIPA buffer (10 mM sodium phosphate pH 7.2, 150 mM NaCl, 1% Na deoxycholate, 0.1% SDS, 1% Nonidet P-40, 2 mM EDTA) supplemented with the Complete protease inhibitor cocktail (Roche Diagnostics). The protein extract (100 μg) was separated by 12% SDS–PAGE, blotted onto a nitrocellulose membrane (Trans-Blot Turbo Transfer System, Bio-Rad Laboratories) monitored by ECL detection (GE Healthcare Life Sciences) with mAb anti-frataxin (18A5DB1 from AbCam) and pAb anti-actinC11 antibodies (sc1615 from Santa Cruz) and imaged with a ChemiDoc XRS system (Bio-Rad Laboratories). Densitometric analysis was performed using the ImageLab 4.1 Software (Bio-Rad Laboratories).

## Results

### Patient’s Description and Mutation Analysis

The FRDA patient, male, was seen at S. Andrea Hospital in Rome (Italy) when he was 29 years old. The patient had a history of alcohol and psychoactive drug abuse since age 17. At the age of 25, he began with gait imbalance, frequent falls and feet paresthesias. Admitted to hospital when 26 years old, the proband presented mild scanning dysarthria, gait and trunk ataxia, dysdiadocokynesia, absent tendon reflexes at upper and lower limbs, impaired position and vibratory sense in lower limbs. Sensory Evoked Potential (SEP) showed a non recordable cortical response at right and a very long latency at left in lower limbs, and cortical response of low amplitude and long latency bilaterally at upper limbs. Brainstem Auditory Evoked Response (BAER) showed long latency wave III and V bilaterally. He presented signs of ventricular hypertrophy at echocardiography and sensory axonal polyneuropathy at Electromyography (EMG). Fundus oculi, cognitive performance, blood glucose and brain MRI were normal. The onset at 25 years of age places this patient at the boundary between a classical and a late onset phenotype (Metz et al., 2013). Alcohol and drug abuse did not thus seem to have a strong impact in determining the age at onset.

Genetic analysis showed that the patient carries a GAA expansion (250 repeats) and a heterozygous G > T mutation in the exon 4 of the *FXN* gene at nucleotide 410 (NM_000144.4:c.410G > T) resulting in a substitution of a highly evolutionary conserved glycine at codon 137 (p.Gly137Val) into a valine. Of the two parents, the father is heterozygous for GAA triplet expansion (250 repeats). The mother is heterozygous for the mutation, the other allele being non-expanded.

### Mapping the Mutation on the Frataxin Structure

**Figure 1:**
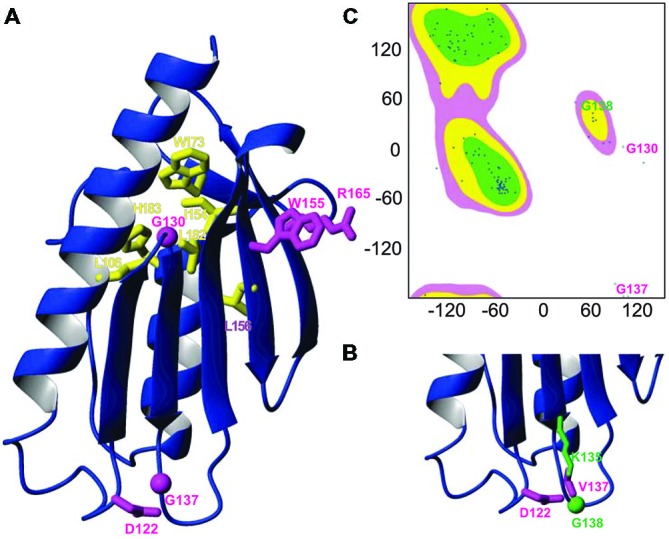
**Mapping the G137V mutation on the frataxin structure. (A)** Ribbon diagram of the human frataxin C-terminal domain (residues 92–210, pdb code 1LY7). The side chains of clinically important mutations are shown explicitly (glycines are identified by spheres). Buried residues which participate to the hydrophobic core are shown in yellow, exposed residues are shown in magenta. **(B)** Zoom on the loop hosting G137 in silico mutated to valine with the surrounding residues N122 and L135, highlighting a possible steric hindrance caused by the mutation. **(C)** Ramachandran plot of the frataxin where glycines 130 and 137 are explicitly marked.

To rationalize the role of the mutation, we mapped it onto the frataxin structure and compared its position with other clinically important missense mutations. Interestingly, G137 maps in the evolutionary conserved C-terminal domain (residues 92–210) as all the other missense mutations, strongly suggesting that this region is the functionally most important region. The C-terminal domain forms a compact globular fold with an alpha-beta structure (Musco et al., 2000). Two helices (95–113 and 182–194) pack against an anti-parallel beta-sheet formed by seven consecutive strands. The known mutations cluster into two families which have interesting commonalities (**Figures 1A,B**). Residues Leu106, Ile154, Leu156, Trp173, Leu182 and His183 are all part of the hydrophobic core and nearly all pack against each other, suggesting that their mutations cause complementary destabilization of the fold. The second family contains exposed residues. Trp155 and Arg165 pack against each other as many tryptophan-arginine pairs do because of the energetic gain of delocalizing pi electrons (Burley and Petsko, 1986). The remaining mutations affect Asp122, Gly130 and Gly137. Gly130 is at the beginning of the β1β2 turn and is a residue in the disallowed region of the Ramachandran plot having both positive phi and psi angles (**Figure 1C**). Gly137 is located at the end of the strand β2, just before Gly138 which has a role similar to Gly130 but in the β2β3 turn. As this residue, Gly138 has also positive phi values. Glycine and valine are both non-polar residues but valine has a bulkier side chain, which could cause steric hindrance with other neighboring residues (e.g., Asp122 and Lys135). Mutation of glycines into any other amino acid will also have an important influence on the turn conformation because of the unique possibility of glycines of adopting positive phi values.

**Figure 2:**
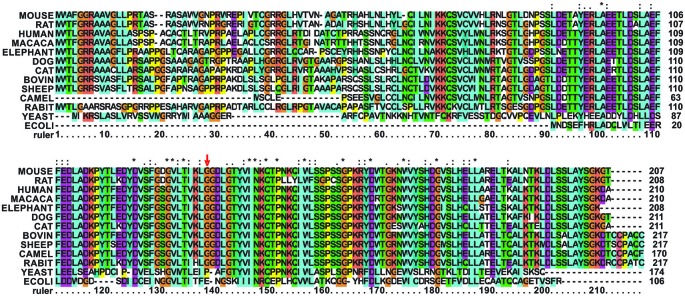
**Multiple alignment of mammalian frataxins compared to the yeast and *E. coli* orthologs**. The position of G137 is marked with a red arrow. G137 and in general the loop residues are conserved in mammalians but not in other species. The alignment was done using the freely available Clustalx software (http://www.clustal.org/). Stars stand for completely conserved residues, colons and dots for decreasing conservation.

Gly137 is not evolutionary conserved as the rest of the turn but present in several eukaryotes. It is replaced by a glutamate in bacterial frataxins (e.g., Glu44 in *E. coli* CyaY) and corresponds to a proline (Pro63) in *S. cerevisiae* frataxin Yfh1 (**Figure 2**).

### The G137V Mutation does not Affect Structure but Strongly Destabilizes the Fold

We then probed *in vitro* the effect of the mutation on the structure of the frataxin variant by producing the human protein by overexpression in bacteria. We used a fragment 90–210 of the protein, rather than the full-length protein, because this already extensively characterized variant spans the evolutionary conserved C-terminal domain which is the only region of the protein with a tertiary fold and the functionally important part of the protein (Musco et al., 2000).

**Figure 3:**
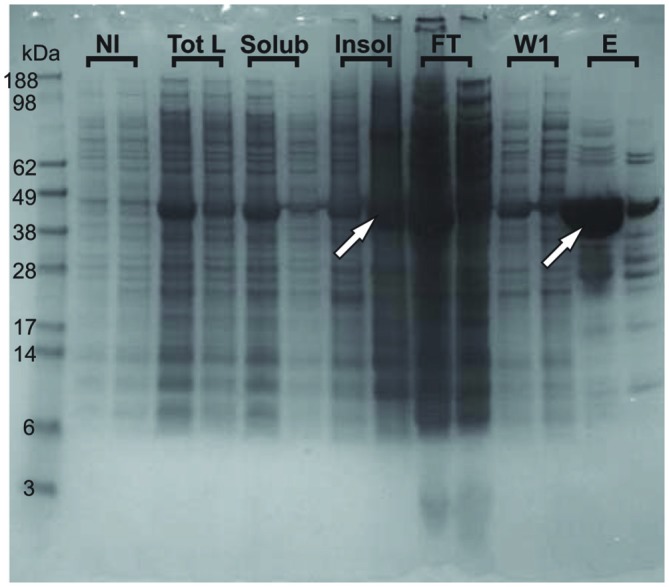
**Tendency of the G137V mutant to form inclusion bodies in bacteria**. The wild-type and G137V mutant are compared on Nu-PAGE MES SDS gel. For each fraction (NI, Tot L, Solub, INSOL, FT, W1 and E), the first sample loaded is wild type and the second is G137V. NI stands for Non-Induced fractions (before induction with IPTG); Tot L: fractions after 3 h-induction with IPTG, Total Lysate; Solub: Soluble fraction of the previous Tot L; INSOL: Insoluble fraction corresponding to the inclusion bodies; FT: Flow Through after first step NI-NTA purification; W1: first Wash; E: Elution with 300 mM Imidazole. The arrows indicate retention of the G137V mutant in inclusion bodies explaining the lower final concentration of proteins obtained in the elution after the first step of purification compared to the wild-type protein.

We could observe an effect of destabilization of the fold already at the level of overexpression. When expressed in *E. coli*, the wild-type protein mostly expresses as a soluble protein. At variance, the G137V mutant is partly expressed in inclusion bodies so that lower amounts of the protein are obtained in the soluble fraction used for the purification (**Figure 3**). A similar behavior was previously observed for other missense mutants (Correia et al., 2008). Although indirect because obtained in a heterologous host, this behavior suggests a lower folding efficiency (Winkelmann et al., 2010).

**Figure 4:**
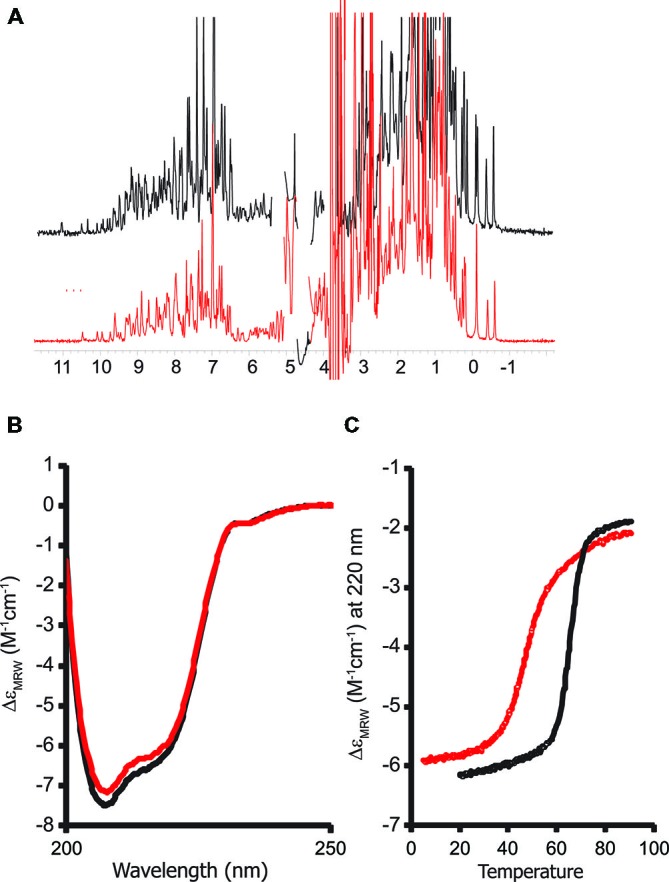
**Probing the structure of the G137V mutant and comparison with the wild-type protein**. The spectra of wild-type human frataxin are plotted in black, those of the mutant in red. **(A)** Comparison of the one-dimensional ^1^H nuclear magnetic resonance (NMR) spectra recorded in H_2_O/D_2_O (90/10 v/v) at pH 8. **(B)** Far-UV circular dichroism (CD) spectra. **(C)** Thermal denaturation scans monitored by far-UV CD. The spectra were recorded at 220 nm and pH 8. CD intensities are presented as the CD absorption coefficient calculated on a mean residue weight basis (δɛ_MRW_).

We recorded a one-dimensional ^1^H NMR spectrum of the purified protein. A wide dispersion of the resonances (**Figure 4A**), especially in the regions of methyl and HN signals (i.e., between 1 and -1 ppm and between 12–7 ppm respectively) is typical of stably folded proteins and proves that the G137V mutant retains its tertiary structure. The spectrum is similar to that of the wild-type protein. Consequently the mutation seems to have no effect on the global fold.

These data were confirmed by CD. The far-UV CD spectrum of the G137V mutant has features very similar to those of the wild-type protein, presenting two minima at ca. 208 and 222 nm that are typical of proteins with an appreciable helical structure content (**Figure 4B**). However, a thermal denaturation scanning followed by CD gave a behavior markedly different from that of the wild-type (Adinolfi et al., 2002; Correia et al., 2006; **Figure 4C**). Thermal unfolding is reversible for both proteins but the melting temperature of the mutant is appreciably lower (46°C and 66°C at pH 8 for G137V and for the wild-type, respectively) which indicates a decrease in stability. Comparison of the melting point with the values previously reported for other clinically important mutants indicates that the effect of this mutation is among the most destabilizing being only marginally higher than the value measured for G130V (43°C; **Table 1**). Interestingly, the two glycine-to-valine mutations of G130 and G137 lead to similar decreases of the fold stability.

**Table 1:** **Comparison of the melting temperatures of the different frataxin variants**.

Variant	pH	Melting temperature
WT	7.9	66.3
D122Y	7.7	50.4
G130V	7.5	43.2
G137V	8.0	46.0
I154F	8.5	50.7
W155R	9.2	61.4

The values for the variants not described in this work are reported by Correia et al. (2006).

### The G137V Mutant Has No Marked Effect on *In Vitro* Iron-Sulfur Cluster Formation

**Figure 5:**
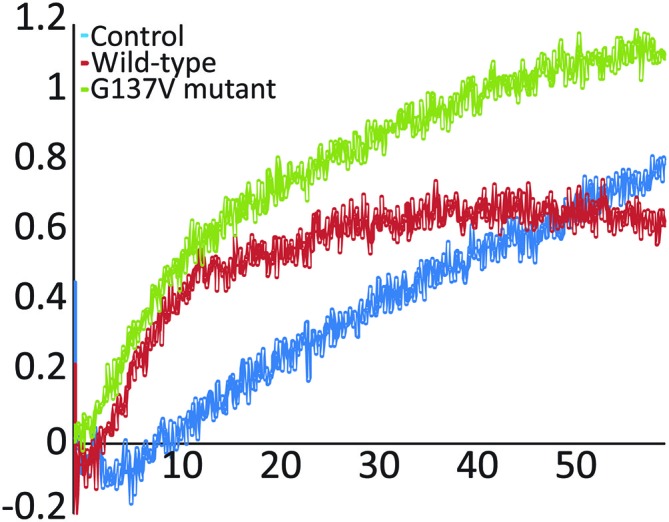
***In vitro* enzymatic reconstitution of iron sulfur clusters**. Absorption spectra were recorded as a function of time in the absence of frataxin and in the presence of wild-type frataxin or the mutants G137V and W155R. The experiments were carried out using 50 μM IscU, 25 μM iron, 250 μM cysteine, 3 mM DTT and 1 μM Nfs1. When present, the frataxin concentration was 50 μM. Only the initial rates were monitored and compared to the control.

Finally, we probed the effect of the mutation on function by following enzymatic iron-sulfur cluster formation *in vitro* in the presence and absence of wild-type and G137V mutated frataxin. In the assay, cysteine is converted into alanine and persulfide by the desulfurase Nfs1/IscS. The persulfide is passed on to the scaffold protein Isu (or IscU in prokaryotes) where, under strict anerobic and reducing conditions, the cluster forms as evidenced by absorbance spectroscopy detected at 400–550 nm (Yang et al., 2006; Adinolfi et al., 2009). It was previously demonstrated that frataxin has a marked effect on this *in vitro* assay which depends on the origin of the desulfurase (Bridwell-Rabb et al., 2012): eukaryotic frataxin is an activator of the enzymatic activity of NFS1 (Tsai and Barondeau, 2010), whereas prokaryotic frataxin acts as a strong inhibitor of bacterial IscS (Adinolfi et al., 2009). *In vivo*, yeast, bacterial and human proteins rescue, albeit not completely, function in knock-out systems (Cavadini et al., 2000; Bedekovics et al., 2007). Interestingly, when we compared the effect of the G130V mutant in the assay with that of wild-type frataxin, we did not observe any marked difference of behavior comparing the effect of wild-type frataxin and the mutant (**Figure 5**) suggesting that mutation has no a direct functional effect atleast under the conditions of this assay.

These results indicate that, while affecting the stability of the protein, the G137V mutation does not significantly impair function, that is, when frataxin is folded, its function is not affected by the mutation.

### The G137V Mutation has Devastating Effects on the Protein Levels

**Figure 6:**
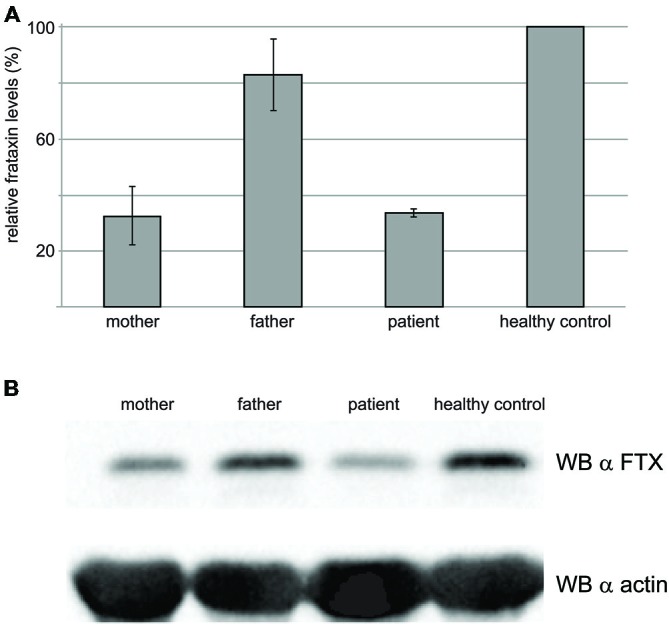
**Western blot results to probe the frataxin levels in the blood. (A)** Total cell extracts were resolved on SDS–PAGE and analyzed with anti-frataxin antibody, or anti-actin, as a loading control. The graph represents relative mean frataxin abundance as quantitated by densitometric analysis of three technical replicates from two different experiments, normalized with actin levels. Error bars represents standard deviation of the mean. **(B)** Representative western blot analysis out of three performed with similar results, is shown.

The frataxin levels were estimated by Western blots on the blood of the proband and compared with those of his parents and of a healthy control (**Figure 6**). As expected, the levels of frataxin of the proband were appreciably lower than those of the control (ca. 50% lower). The levels in the heterozygous father are comparable to those of the control. Surprisingly, the mother’s levels were also low and comparable to those of the proband despite the fact that the mother heterozygote for the point mutation and a normal allele is not affected by FRDA. However, it is worth mentioning that the mother had been administered chemiotherapy as a cancer treatment.

## Discussion

Although FRDA is mainly caused by partial impairment of frataxin expression (Campuzano et al., 1996), 4% of FRDA patients are heterozygous, presenting a triplet expansion on one allele and a deleterious point mutation on the other allele. About 20 different point mutations are currently known (Musco et al., 2000; Correia et al., 2006), some of which accounting for atypical clinical presentations. Contrary to the common GAA expansion that alters the levels of protein expression, FRDA point mutations are usually thought to be associated with a reduced function of the expressed protein and are thus interesting because they may help us to establish causal linkages between genetic variations and biological phenotypes.

Here, we analyzed the effects of a mutation found in a heterozygote patient carrying a moderate expansion on one allele and a point mutation G137V on the other. During the refereeing process, it was brought to our attention that, interestingly, this mutation had already been reported independently (Costantini et al., 2013). The patient’s age at disease onset and progression are similar to those reported here, supporting a causative relationship between this mutation and the ataxic phenotype. To rationalize the effect of the G137V mutation and understand the mechanism which leads to disease, we analyzed the consequence of the mutation on protein structure, stability and function of human frataxin *in vitro*. The mutant protein is correctly folded and fully functional in our assays even though when put into the context of other mutations, G137V seems to have a strong effect on destabilization. We observed a substantial decrease in the thermodynamic stability of the G137V variant during thermal unfolding as compared to most of the other mutations (wild type > W155R > I154F > D122Y > G137 > G130V), although unfolding is reversible in all cases under the conditions of the experiment. This result can be rationalized by considering that in the frataxin 3D structure, G137 is in the turn between the β2 and β3 strands. It is an exposed residue conserved in mammalian frataxins. Glycines are important residues for the unique properties of their energy landscapes which allow these residues to adopt backbone conformations also in the disallowed region of the Ramachandran plot. For this reason, they are often hosted in loops where they have the important role of providing entropic tension that is essential for folding (Thomas, 1990). Any substitution will necessarily lead to destabilization of the protein. On this account, it is easy to understand the similar destabilizing effect observed for G130V and G137V. Interestingly, in the 3D structure, Asp122, another residues involved in a point mutation of clinical importance, packs against Gly137 suggesting a correlated effect of the two mutations.

Having carried out both a Western blot analysis and biophysical studies, we clearly observed an effect on the protein levels but not on function. In other words, when folded, the mutated frataxin is fully functional but the lower stability makes less efficient the protein folding process and/or may enhance degradation. Accordingly, G137V, like other mutants, tends to form inclusion bodies when overexpressed in bacteria, a feature that is not typical of the wild-type protein and that was proposed to be revealing of an intrinsic tendency to aggregate (Winkelmann et al., 2010).

This may result in lower than normal concentrations of frataxin as supported by evidence that the frataxin levels in the patient described here are below the normal ones. This reduction in protein levels was also identified in the healthy mother of the subject suggesting that most of the reduction in frataxin levels could be due to the G137V mutation and not due to the GAA expansion, the mutation carried by the father, although the father’s expansion seems to determine the age of onset. We thus propose that the mechanism acting in some FRDA heterozygous patients relies on the consequence of a structural impairment which we will call “loss of folding efficiency” to be distinguished from loss/gain of function mechanisms. Although quite different from other neurodegenerative diseases due to toxic aggregation, this mechanism may be important in most of the heterozygous patients and may be relevant also in otherdiseases.

These considerations might suggest a possible therapeutic application for selective treatment of patients having frataxin mutations with reduced folding stability. It is known that the function of some thermodynamically unstable mutant proteins can be recovered in the presence of chemical chaperones (i.e., low molecular weight compounds, such as the cellular osmolytes glycerol and trimethylamine N-oxide), that are able to stabilize nonspecifically the native conformation of many proteins, or by the presence of ligands and inhibitors that specifically bind to a protein, so-called pharmacologic chaperones (Bernier et al., 2004; Atanasov et al., 2007). In this context, it is also important to mention that prove-of-principle marketed point-mutation targeting drugs already exist for other proteins (e.g., Ivacaftor was developed for specific treatment of cystic fibrosis patients carrying G551D mutation; Kapoor et al., 2014).

It remains however to explain the condition of the patient given that, overall, the level of frataxin observed is within that of healthy carriers and comparable to that of the heterozygous mother carrying the G137V mutation. Consistently, the severity of this patient’s status is relatively mild. It should be considered that compound heterozygous patients generally have lower levels of frataxin than homozygous for expansion but the precise levels vary dramatically between tissues (Lazaropoulos et al., 2015). Start codon mutations lead for instance to low levels of frataxin in buccal cells but preserve immunoreactive frataxin levels in blood (Lazaropoulos et al., 2015). Furthermore, peripheral frataxin levels reflect disease features in FRDA but the interpretation depends strongly on the specific mutation. Another level of complexity comes also from the possibility that the personal history of alcohol and drug abuse of this patient could have contributed if not to the age of onset, which is in the average, to the worsening of his symptoms even though the frataxin levels are not in the pathologic range.

Overall, our study thus contributes to show the complexity of correlating the frataxin levels to disease severity and the difficulties of providing a unique prediction of disease onset and progression in heterozygous FRDA patients.

## Funding

AP was funded by MRC (the PF7 consortia EFACTS and SARCOSI) and EU (SARCOSI consortium). SF and RT were funded by Telethon (Grant GGP11102). NF was supported by ESBS (Strasbourg).

## Conflict of Interest Statement

The authors declare that the research was conducted in the absence of any commercial or financial relationships that could be construed as a potential conflict of interest.
